# COVID-19 CT image segmentation method based on swin transformer

**DOI:** 10.3389/fphys.2022.981463

**Published:** 2022-08-22

**Authors:** Weiwei Sun, Jungang Chen, Li Yan, Jinzhao Lin, Yu Pang, Guo Zhang

**Affiliations:** ^1^ Chongqing University of Posts and Telecommunication, Chongqing, China; ^2^ School of Medical Information and Engineering, Southwest Medical University, Luzhou, China

**Keywords:** COVID-19, CT image, deep learning, detection and recognition, lesion segmentation

## Abstract

Owing to its significant contagion and mutation, the new crown pneumonia epidemic has caused more than 520 million infections worldwide and has brought irreversible effects on the society. Computed tomography (CT) images can clearly demonstrate lung lesions of patients. This study used deep learning techniques to assist doctors in the screening and quantitative analysis of this disease. Consequently, this study will help to improve the diagnostic efficiency and reduce the risk of infection. In this study, we propose a new method to improve U-Net for lesion segmentation in the chest CT images of COVID-19 patients. 750 annotated chest CT images of 150 patients diagnosed with COVID-19 were selected to classify, identify, and segment the background area, lung area, ground glass opacity, and lung parenchyma. First, to address the problem of a loss of lesion detail during down sampling, we replaced part of the convolution operation with atrous convolution in the encoder structure of the segmentation network and employed convolutional block attention module (CBAM) to enhance the weighting of important feature information. Second, the Swin Transformer structure is introduced in the last layer of the encoder to reduce the number of parameters and improve network performance. We used the CC-CCII lesion segmentation dataset for training and validation of the model effectiveness. The results of ablation experiments demonstrate that this method achieved significant performance gain, in which the mean pixel accuracy is 87.62%, mean intersection over union is 80.6%, and dice similarity coefficient is 88.27%. Further, we verified that this model achieved superior performance in comparison to other models. Thus, the method proposed herein can better assist doctors in evaluating and analyzing the condition of COVID-19 patients.

## 1 Introduction

According to several studies (Zhu et al., 2019; Dong et al., 2020; Xu et al., 2020), computed tomography (CT) clearly displays the characteristic lung lesions of Covid-19 in patients. However, CT scans contain hundreds of slices, and CT images must be reconstructed and transmitted through an image archiving and communication system for doctors to interpret results and diagnose patients. Covid-19 and other types of pneumonia are generally identified by radiologists by simply processing images at communication system terminals, reading them, or projecting them through a lamp (Bai et al., 2020; Song et al., 2021; Bernheim et al., 2020; Rubin et al., 2020; Wong et al., 2020; Lee et al., 2001). Simultaneously, radiologists must be experienced to achieve sufficient detection results. Covid-19 has similar medical imaging characteristics to other types of pneumonia (Shi et al., 2020), and CT can be used to determine whether a patient is infected with viral pneumonia (Covid-19 is a viral pneumonia caused by the SARS-COV-2 virus) (Chen and Li, 2020). However, CT is unable to determine which virus causes viral pneumonia; the novel coronavirus or another virus, making it difficult to distinguish and diagnose the virus type. Considering these difficulties, quickly and accurately distinguishing between Covid-19 and other types of pneumonia is crucial to facilitating the screening process in clinical practice. Therefore, with the AI-assisted diagnosis of medical images, accurate and efficient recognition of Covid-19 lung CT images is of profound significance for controlling the epidemic (Ardila et al., 2019; Esteva et al., 2019; Esteva et al., 2017; Litjens et al., 2017; Mei et al., 2020; Qin et al., 2019; Topol 2019; Li et al., 2020; Jaiswal et al., 2020).

Locality is used by traditional convolutional networks to improve efficiency but at the cost of losing the connection in a global context. Convolutional architecture has an inherent induction bias, lacking an understanding of position dependence in images (Wang et al., 2020; Valanarasu et al., 2021). In a study by Dosovitskiy et al. (2020), the proposed vision transformer (ViT) was trained on large image datasets using location-embedded two-dimensional (2D) image patches as input sequences, thus achieving a performance comparable to that of convolutional networks. Based on the transformer architecture, a self-attention mechanism was utilized to encode the position dependence at a distance to learn efficient representations. However, most existing transformer-based network architectures require large datasets for training. Generalization may be inadequate if the training is performed using insufficient data. In a study by Hassani et al. (2021), a compact convolutional transformer (CCT) (Wang et al., 2021) was proposed to eliminate the misunderstanding of the requirement of a transformer for large amounts of data. It achieves comparable performance on small datasets; however, when the input dimension is large, the operational cost of the self-attention mechanism increases significantly. Global pooling does not use the location information in the process of extracting pneumonia symptoms, potentially causing loss of location information. For imaging tasks, it is important to obtain the spatial position structure of an image.

Therefore, we use a new method to solve the above problems in CT lesion segmentation of COVID-19. To solve the problem of detail loss, we add CBAM (Woo et al., 2018) and atrous convolution to the U-Net encoder part, and replace the partial convolution operation with the empty convolution operation. This can solve the problem of feature image detail loss caused by the decrease of resolution after repeated down-sampling operations. A Swin Transformer (Liu et al., 2021) is added to obtain local information in the CNN network, and the joint loss function is used for optimization during training. Thus, the segmentation of background regions, lungs, ground-glass opacities, and lung parenchyma in the chest CT images of patients is achieved. The results of ablation experiments demonstrate that this method achieved significant performance gain, in which the mean pixel accuracy is 87.62%, mean intersection over union is 80.6%, and dice similarity coefficient is 88.27%. The feasibility and effectiveness of this method are proved. Chest CT examination has a very important application prospect in clinical observation of treatment effect, monitoring of lesions and follow-up.

## 2 Materials and methods

**FIGURE 1:**
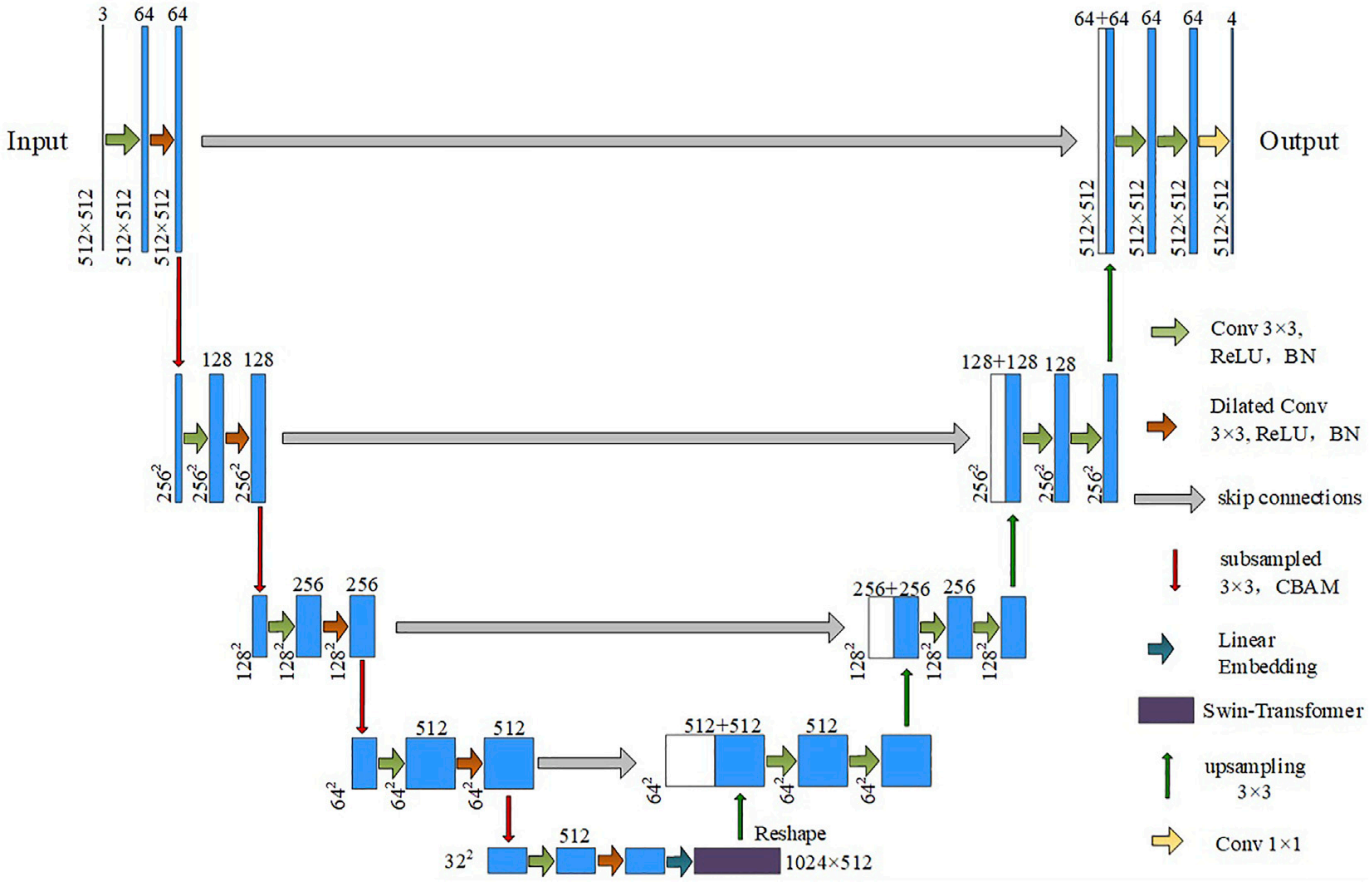
Segmentation network structure diagram.

Here, a new lesion segmentation method in chest CT images of COVID-19 patients is proposed, and the network structure is shown in Figure 1. The input is downsampled 4 times in total. The encoder performs a normal convolution and a dilated convolution operation before downsampling. The BN layer and the activation function layer are added to speed up the network convergence process. The CBAM mechanism is introduced in the downsampling process. After each downsampling iteration, the size of the feature vector is halved, and the number of channels is doubled. In the experiment, images with a height and width of 512 and three channels are used as input, that is,512 × 512 × 3 After being processed by the encoder part, a feature vector of size 32 × 32 × 512 is output. Then, the downsampled feature images are flattened to fit the vector dimension of the Swin Transformer structure by linear embedding. The vector dimension does not change in the Transformer encoder structure, and a sequence vector of 1,024 × 512 dimensions is output. The sequence vector is restored to 32 × 32 × 512 by the Reshape operation to fit the input dimension requirement of the segmentation network upsampling. Finally, the segmentation result whose height and width are consistent with the input is obtained after passing through the decoder for four upsampling iterations.

### 2.1 Convolution attention module

**FIGURE 2:**
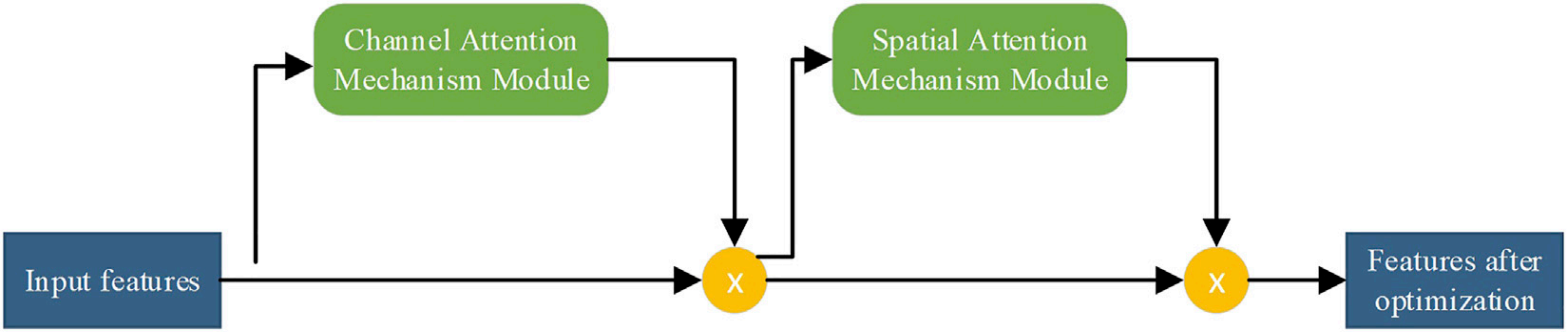
Flowchart of the attention module of the convolutional block.

We use an attention mechanism in the network to perform weight adjustment on the feature vectors. This is similar to how the human brain focuses on important information. Important information is made more prominent, and other information is filtered. The convolutional attention module is composed of channel attention and spatial attention modules, which are used for the attention mechanism of the feature vector channel and space, respectively. The process is shown in Figure 2. Finally, the attention weights are multiplied by the input feature image to obtain the output feature image.

#### 2.1.1 Channel attention mechanism

**FIGURE 3:**
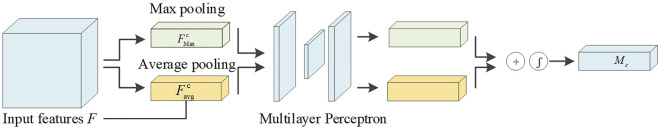
Flowchart of channel attention mechanism.

Channel attention assigns weights to each channel of the feature image. Valid channel weights are increased, and invalid channel weights are suppressed. The flow of the channel attention mechanism is shown in Figure 3. The input feature $F\in {\mathbb{R}}^{H\times W\times C}$ is average-pooled to generate the vector $F_{\text{avg}}^{\text{C}}\in {\mathbb{R}}^{1\times 1\times C}$ , where C represents the channel. The vector $F_{\text{max}}^{\text{C}}\in {\mathbb{R}}^{1\times 1\times C}$ is generated by a max-pooling operation. Average pooling has the advantage of optimizing the spatial information of feature images. Max pooling can extract landmark information in feature images. The two output features are fed into a shared multilayer perceptron, and features with contextual descriptions are generated. Finally, the ReLU activation function is used to output the feature image channel weights. Feature images are summed and merged elementwise. The feature vector $M_{\text{C}}\in {\mathbb{R}}^{C\times 1\times 1}$ is output through the sigmoid activation function.

According to the above process, the calculation formula is as follows:

$$\begin{array}{l} M_{c}\left(F\right)=\sigma \left(\mathrm{MLP}\left(\mathrm{AvgPool}\left(F\right)\right)+\mathrm{MLP}\left(\mathrm{MaxPool}\left(F\right)\right)\right)\\ \;\;\;\;\;\;\;\;\;\;\;\;\;\;\;\;=\sigma \left(W_{1}\left(W_{0}\left(F_{\text{avg}}^{\text{c}}\right)\right)+W_{1}\left(W_{0}\left(F_{\text{max}}^{\text{c}}\right)\right)\right) \end{array}$$ (1)

where σ represents the Sigmoid activation function, AvgPool represents the average pooling operation, MaxPool represents the maximum pooling operation, MLP represents the shared multi-layer perceptron, and $W_{0}$ and $W_{1}\in {\mathbb{R}}^{C/r\times C}$ are the weights of the shared multi-layer perceptron.

#### 2.1.2 Spatial attention mechanism

**FIGURE 4:**
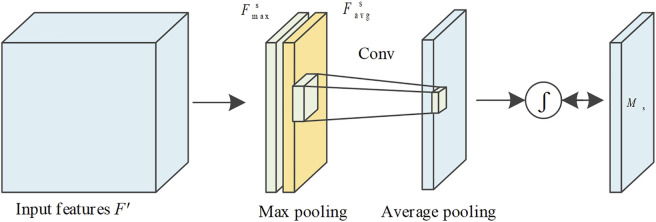
Flow chart of spatial attention mechanism.

The spatial attention mechanism can measure some regions of the feature image to obtain higher responses, and the mechanism flow is shown in Figure 4. Suppose the feature vector optimized by the channel attention module is $F^{\prime }\in {\mathbb{R}}^{H\times W\times C}$ . $F^{\prime }$ generates the two-dimensional vector $F_{\text{avg}}^{\text{S}}\in {\mathbb{R}}^{H\times W\times 1}$ by the max pooling operation, and $F_{\text{max}}^{\text{S}}\in {\mathbb{R}}^{H\times W\times 1}$ is generated through average pooling, where S represents a channel. The two-dimensional vector information obtained by the pooling operation is concatenated. The feature information is fused through the convolution operation, and a two-dimensional spatial attention image is generated through the sigmoid activation function. Finally, the output of the spatial attention module is dot multiplied with the feature image at the pixel level to obtain the weighted feature image.

The equation of the above process is as follows:

$$\begin{array}{l} M_{s}\left(F\right)=\sigma \left(f^{7\times 7}\left(\left[\mathrm{MaxPool}\left(F\prime \right),\mathrm{AvgPool}\left(F\prime \right)\right]\right)\right)\\ \;\;\;=\sigma \left(f^{7\times 7}\left(\left[F_{\text{max}}^{s},F_{\text{avg}}^{s}\right]\right)\right) \end{array}$$ (2)

where σ is the sigmoid activation function, and $f^{7\times 7}$ indicates that the feature vector in parentheses is convolved with a convolution kernel of size 7 × 7.

F represents the feature image, the output $F^{\prime }$ is optimized by the channel attention module, and the output $F^{\prime \prime }$ is optimized by the spatial attention module. Therefore, feature F is optimized by the CBAM module:

$$F^{\prime }=M_{c}\left(F\right)⊗F$$ (3)

$$F^{\prime \prime }=M_{s}\left(F‘\right)⊗F^{\prime }$$ (4)

where ⊗ represents that the elementwise multiplication.

### 2.2 Atrous convolution

**FIGURE 5:**
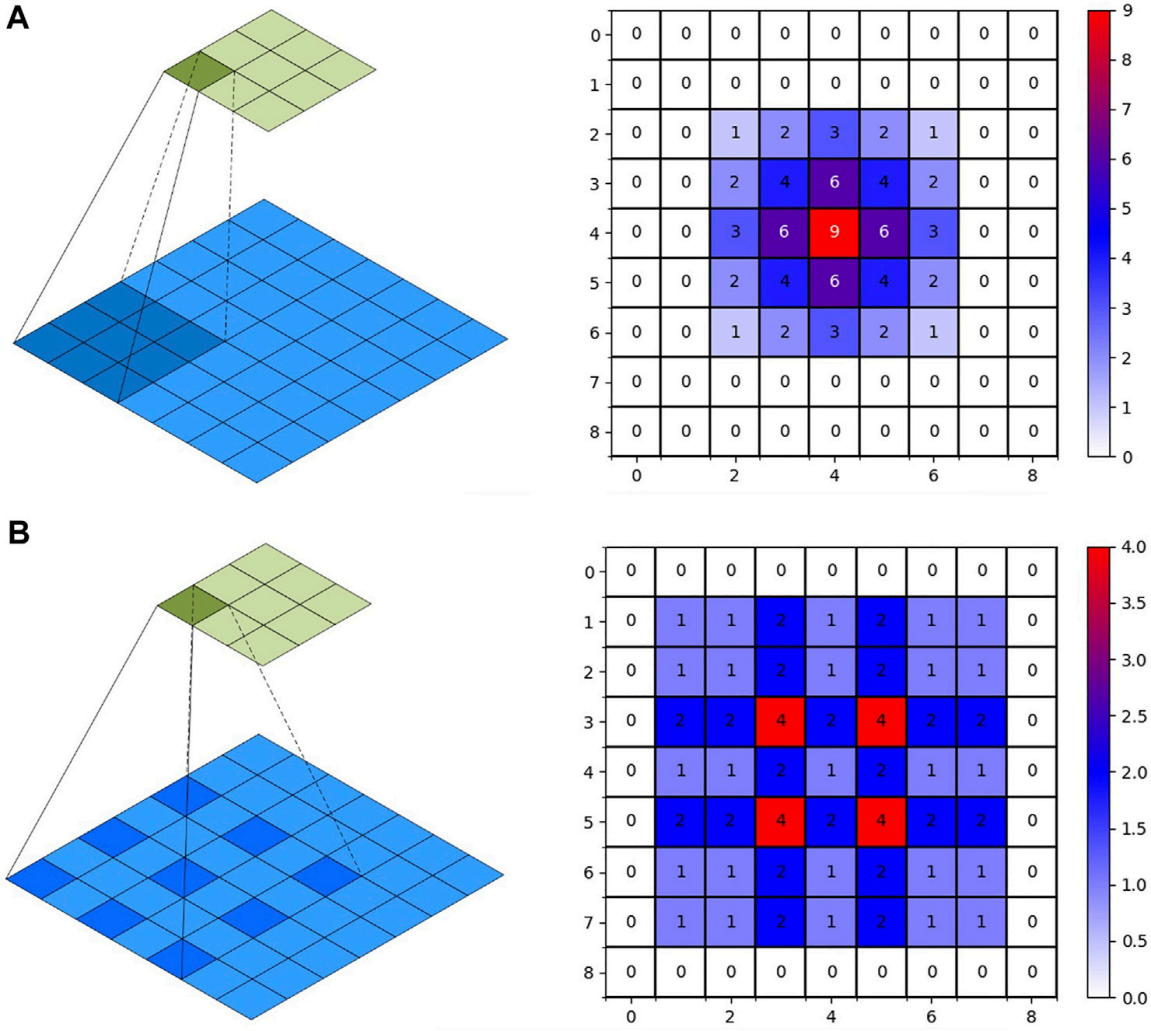
Convolution operation. **(A)**: 2D-Convolution. **(B)**: Dilated Convolution.

Feature information is extracted using U-Net model convolution operations. Due to device performance limitations, multiple pooling operations reduce the resolution of feature vectors. When using the convolution operation to extract higher-level features, the next convolution operation can obtain a larger receptive field. However, as the feature size decreases, feature information will be lost. The restoration detail information cannot be restored, while upsampling restores the size. Replacing ordinary convolution operations with atrous convolution can achieve a larger receptive field range within a limited convolution kernel. Therefore, the loss of detail information caused by the downsampling process can be solved. The ordinary convolution and atrous convolution methods and the obtained receptive fields are shown in Figure 5. In the right image of Figures 5A,B, the feature maps of 9 × 9 use a convolution kernel of size 3 × 3 and stride 1 for convolution operation. In the right picture of Figure 5A, the receptive field is obtained after two ordinary convolution iterations; the range is 5 × 5. In the right picture of Figure 5B, the receptive field is obtained after one ordinary convolution and one dilated convolution with a dilation factor of 2; the range is 7 × 7. It shows that a larger receptive field range is obtained after using atrous convolution. The numbers in the figure represent the number of times the pixels are convolved.

When using continuous atrous convolution, the dilation factor cannot be a common divisor greater than 1. And the expansion factor must satisfy the following formula:

$$M_{i}=\max\left[M_{i+1}-2r_{i},M_{i+1}-2\left(M_{i+1}-r_{i}\right),r_{i}\right]$$ (5)

where $M_{i}$ represents the maximum expansion factor of the i-th layer, and $r_{i}$ is the expansion factor that represents the distance between adjacent elements in the hollow convolution kernel, which should be less than or equal to the size of the convolution kernel. In the atrous convolution operation, the convolution kernel size is fixed. When the dilation rate increases, the spacing of adjacent elements in the convolution kernel increases. It is also possible to keep the height and width of the original input feature map unchanged.

### 2.3 Swin transformer module

After improving the convolutional structure network of U-Net to extract feature information, we use the Swin Transformer to extract the global information from the feature information. We combine the CNN with the Transformer structural model. The insufficiency of context dependencies in the acquisition of low-level features by convolutional networks will be compensated. Compared with ViT, we improve the Transformer encoder by introducing windows multi-head self-attention (W-MSA) and shifted windows multi-head self-attention (SW-MSA) (Hatamizadeh et al., 2022). Assuming the input is $x_{l-1}$ , the formula is as follows:

$${x^{\prime }}_{l-1}=x_{l-1}+W-\mathrm{MSA}\left(\mathrm{LN}\left(x_{l-1}\right)\right),$$ (6)

$$x_{l}={x^{\prime }}_{l-1}+\mathrm{MLP}\left(\mathrm{LN}\left({x^{\prime }}_{l-1}\right)\right),$$ (7)

$${x^{\prime }}_{l}=x_{l}+\mathrm{SW}-\mathrm{MSA}\left(\mathrm{LN}\left(x_{l}\right)\right),$$ (8)

$$x_{l+1}={x^{\prime }}_{l}+\mathrm{MLP}\left(\mathrm{LN}\left({x^{\prime }}_{l}\right)\right).$$ (9)

where l∈{1,2,⋯,L}

**FIGURE 6:**
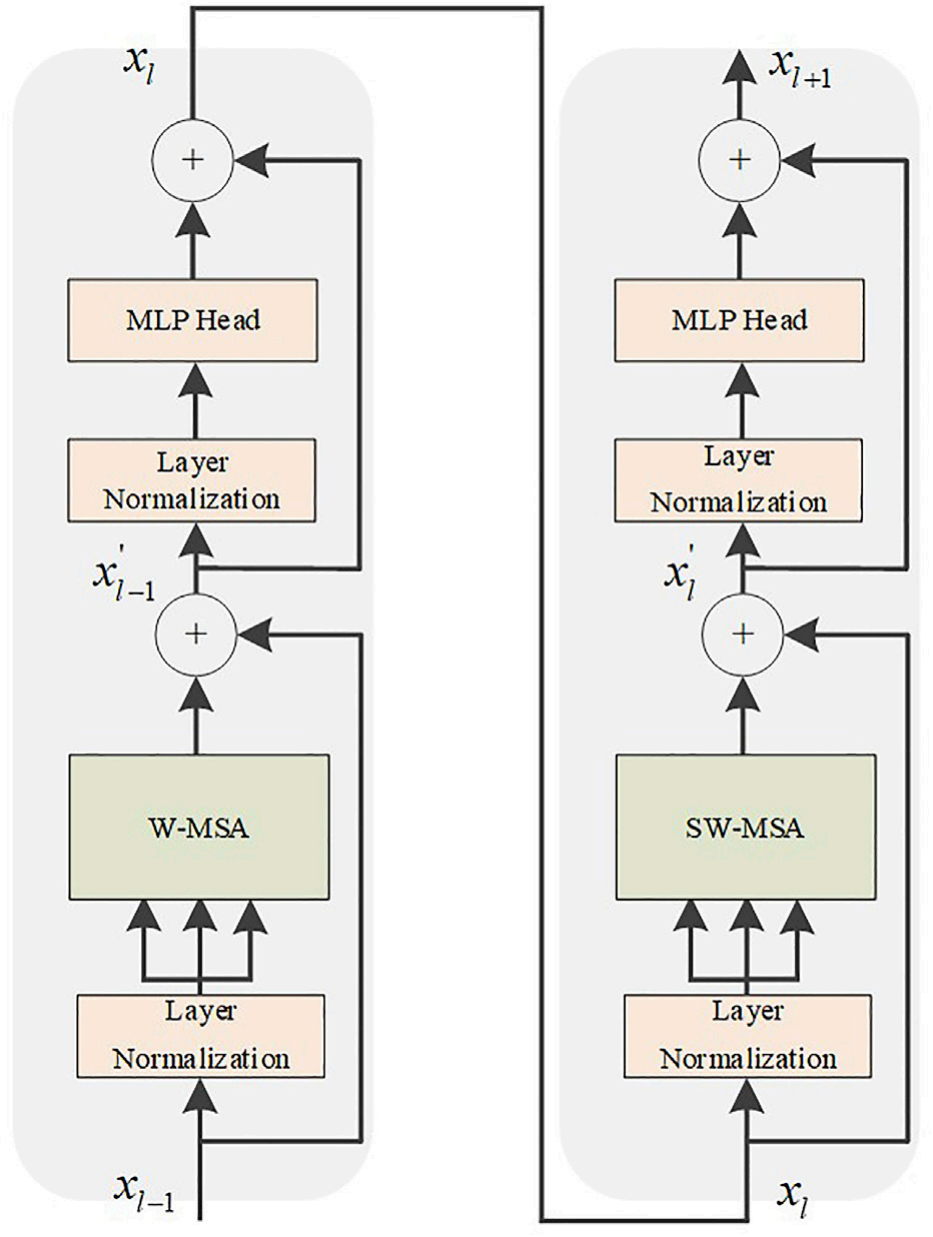
Swin Transformer module.

According to the Swin Transformer formula, it can be concluded that the structure consists of two Transformer encoder modules. After the input is normalized by the layer, the attention value is calculated using W-MSA, and the residual structure is formed with the original input. After layer normalization and MLP operation, the encoder module with the SW-MSA calculation method is used to output the feature vector. The Swin Transformer structure is shown in Figure 6. Compared with the ViT, an encoder module is added, and the redesigned W-MSA and SW-MSA calculation methods greatly reduce the computational complexity.

**FIGURE 7:**
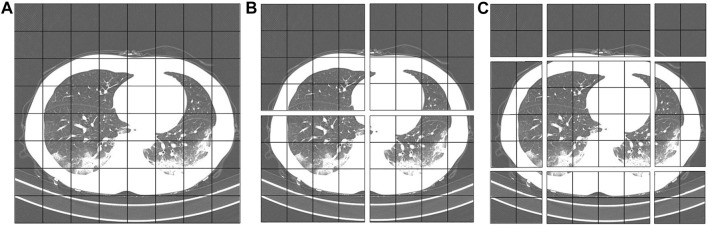
Operation diagram of self-attention mechanism. **(A)**: MSA. **(B)**: W-MSA. **(C)**: SW-MSA.

In the W-MSA operation of the Swin Transformer, the feature map is divided into windows of the same size, which is equivalent to reducing the size of the patch. Thereby the computational complexity is reduced. We utilize the same self-attention mechanism as ViT inside each individual window. However, after dividing the feature map into separate feature windows, the attention mechanism values of the feature windows are calculated separately, and there is no information interaction between them. As a result, the self-attention mechanism cannot obtain global information. Therefore, the SW-MSA operation is increased, and the window operation is shifted. This solves the defect that information cannot be exchanged between W-MSA operation windows. The operation flow of common MSA, W-MSA, and SW-MSA is shown in Figure 7. The W-MSA window size is 4. In the SW-MSA operation, the feature window is divided into three different patch sizes, which are 2 × 2, 2 × 4, and 4 × 4 sizes. After combining four 2 × 2-sized windows and combining four 2 × 4-sized windows, two feature windows with patch size 2 × 2 are obtained. Then, the attention value is obtained by continuing the calculation of W-MSA. Finally, the original window dimensions are restored. As such, not only is the computational complexity reduced, but the interactive information between the windows can be obtained.

### 2.4 Optimization of loss function

**FIGURE 8:**
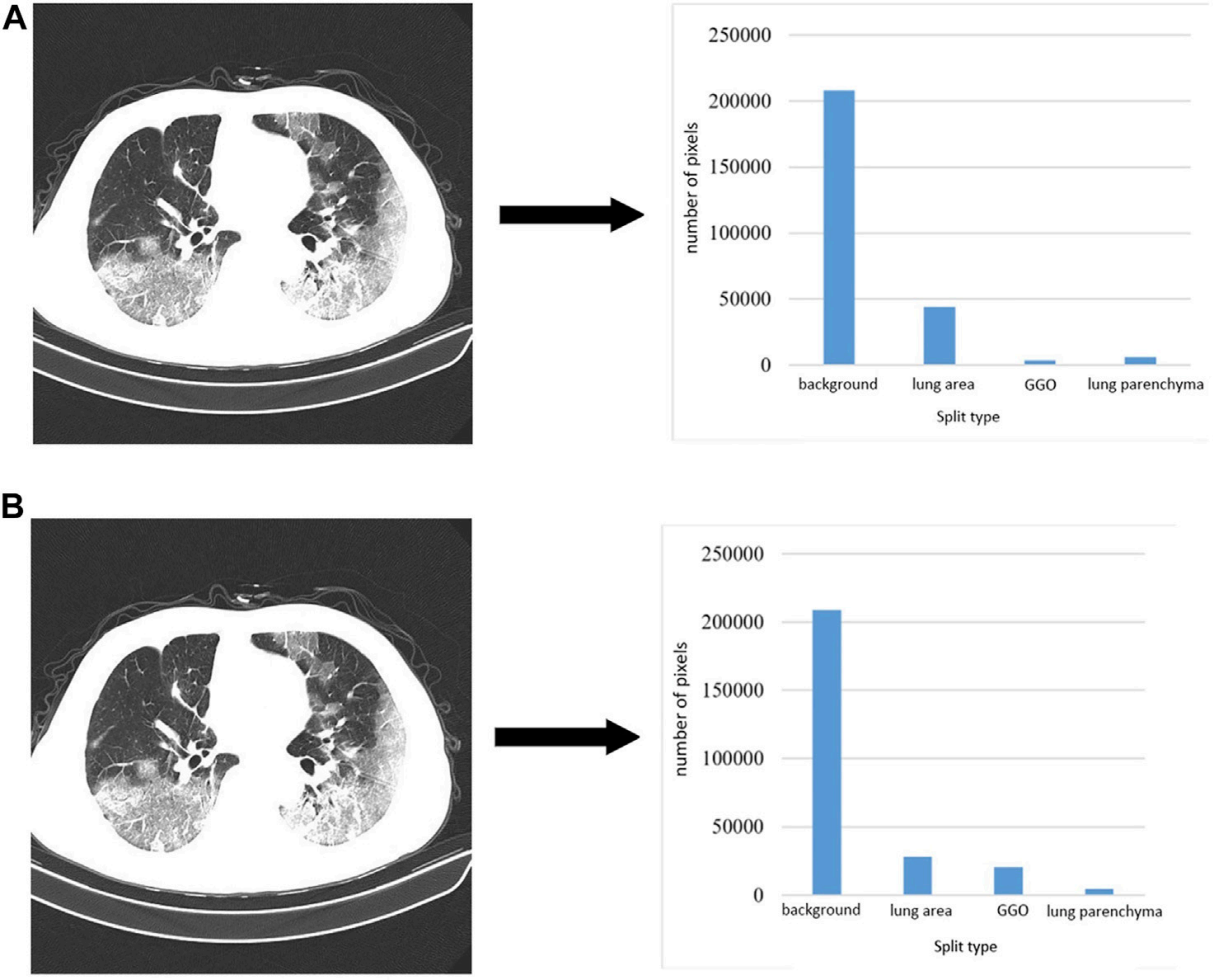
CT images and corresponding pixel category distributions.

In medical image segmentation, common loss functions include cross entropy loss (CE loss) and dice coefficient loss (Dice loss). The chest CT image segmentation method we proposed includes four categories: background region, lung region, ground glass opacity, and lung parenchyma. Figure 8 shows the chest CT images and the pixel distribution maps of different categories in the corresponding segmentation gold standard. The abscissa is the segmentation type, and the ordinate is the number of pixels. It can be seen that the proportion of ground glass and lung parenchyma is much smaller than the background and lung areas. This is common in mild and moderate patients, and there may even be no focal manifestations. Therefore, uneven data distribution will be caused in the experiment, which makes network training more difficult.

The cross-entropy loss function compares the pixel-predicted value output by the training model with the real value. In the case of training without overfitting, the smaller the loss value, the better the result. The formula is as follows:

$$CE\;loss=-y^{*}\log_{2}\left(y^{\prime }\right)$$ (10)

where *y* is the real label paper, $y^{\prime }$ is the predicted value, and the loss function has the same prediction weight for each category. As shown in Figure 8, the background area accounts for a large proportion, and the factors leading to the final result will be biased towards the background area. After training, the performance value of the loss function is small, but it cannot reflect the classification effect of other categories through the loss value.

In dice loss, dice represents the dice similarly coefficient (DSC), which indicates the degree of similarity between two sample areas; the value is between 0 and 1; the larger the value, the higher the similarity. Assuming that A and B represent sets of regions, the DSC formula is as follows:

$$Dice=\frac{2\left|A\cap B\right|}{\left|A\right|+\left|B\right|}$$ (11)

where ∩ represents the intersection of sets, and the dice loss formula can be obtained according to the DSC. The formula is as follows:

$$Diceloss=1-\frac{2\left|Y\cap \bar{Y}\right|+1}{\left|Y\right|+\left|\bar{Y}\right|+1}$$ (12)

where Y is the real segmentation area, and $\bar{Y}$ is the model prediction area. We add 1 to the denominator and numerator to prevent the denominator from being zero and to reduce the possibility of overfitting during the training process. Compared with the CE loss function, dice loss is not affected by the background when the number of pixel categories is unevenly distributed. However, training instability occurs when the prediction is incorrect. Therefore, we combine the CE loss function and dice loss as a joint loss function and use the CE loss function to guide dice loss for training. The formula is as follows:

loss=0.5×CE+Dice loss (13)

$$loss=0.5\times Y\cdot \log_{2}\left(\bar{Y}\right)+1-\frac{2\left|Y\cap \bar{Y}\right|+1}{\left|Y\right|+\left|\bar{Y}\right|+1}$$ (14)

### 2.5 Datasets

**FIGURE 9:**
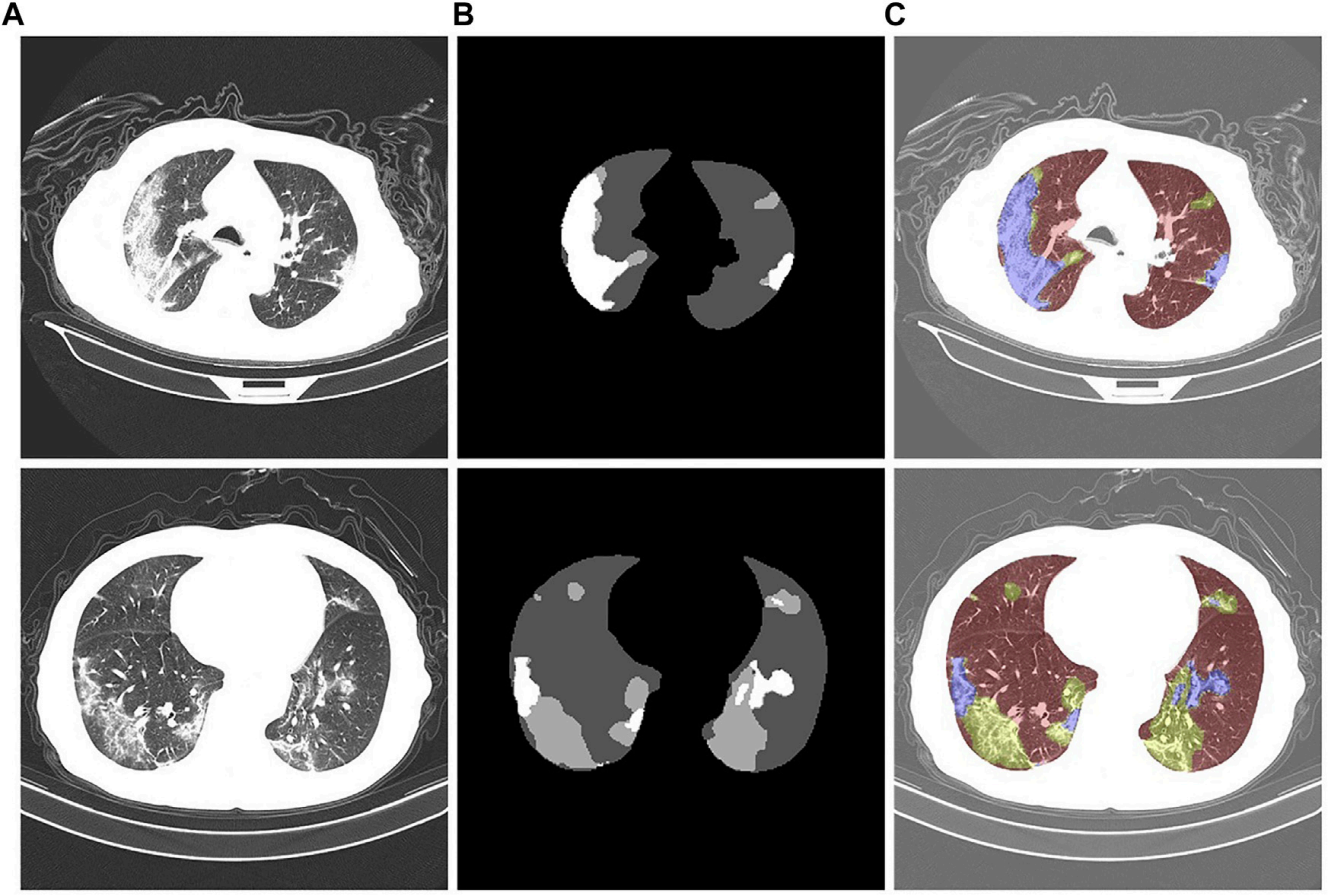
Chest CT images of COVID-19 patients. **(A)**: Initial CT image. **(B)**: Gold standard. **(C)**: Color-annotated segmentation results.

We utilized a dataset from the China Consortium for Chest CT Imaging Research (CC-CCII) (Ai et al., 2020). The CC-CCII dataset contains 617,775 CT images from 6,752 CT scans of 4,154 patients. The study sample size was estimated by standard AI training and validation methods. Patients were randomly assigned to a training set (60%), an internal validation set (20%) or a test set (20%). We chose to use 750 annotated chest CT images selected from 150 COVID-19 patients by five radiologists with 15 years of experience. These images include background areas, lung areas, ground-glass opacities, and lung parenchyma. Mild patients mainly present with ground-glass opacity, which is distributed in the lower lobes of both lungs and adheres closely to the pleura. Ground-glass shadows are characterized by spreading toward the center and blurring at the edges. In moderate patients, the number of lesions proliferated, and the lesions were markedly plaque-like. The patient is accompanied by a condition of cough and fatigue. In severe patients, the density of lung tissue increases and the lung parenchyma changes. The patient presented with fever and headache. An example of the segmentation of a chest CT image of a COVID-19 patient is shown in Figure 9. Figure 9A is the initial image, Figure 9B is the gold standard of the dataset, and Figure 9C is after the gold standard mask and the initial CT image are superimposed; the highlighted color is used to distinguish the segmentation results. The gray area is the background area of the patient, the red area is the lung area, the yellow area is the ground glass opacity, and the blue area is the lung parenchyma.

#### 2.5.1 Data augmentation

**FIGURE 10:**
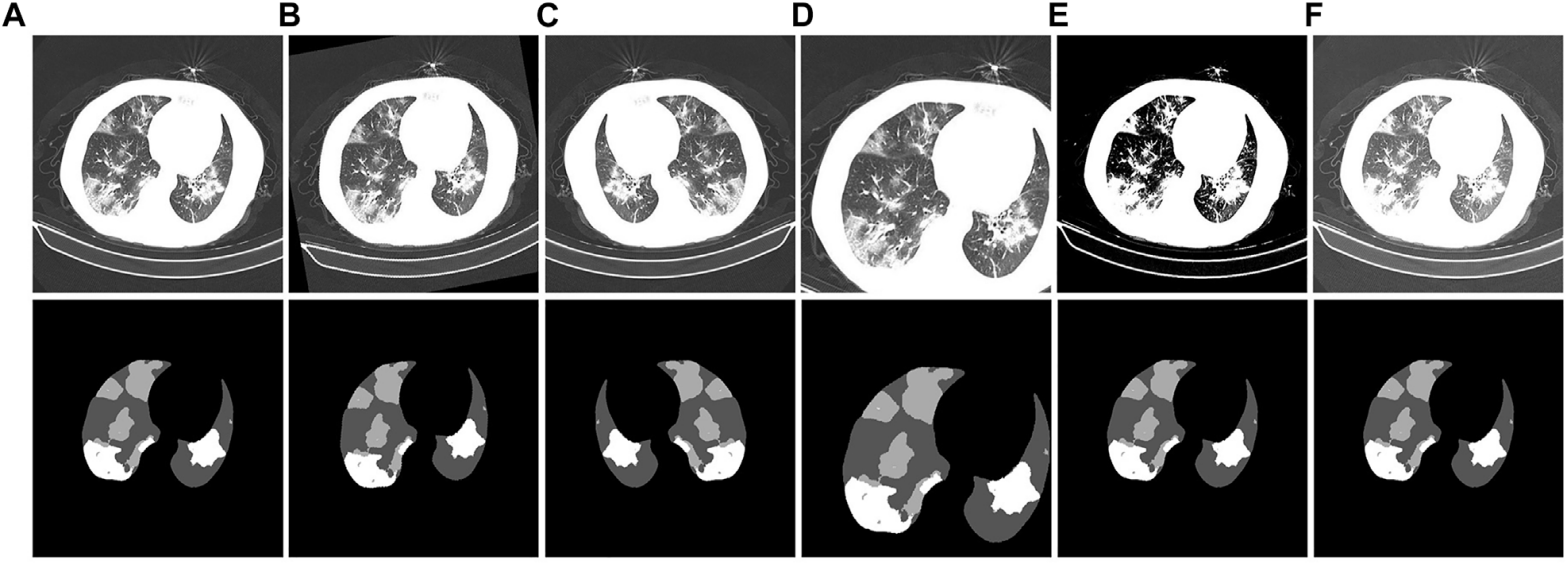
Example of CT image data-enhancement results. **(A)**: the original CT image and its corresponding gold standard. **(B)**: the CT image obtained after rotation and its corresponding gold standard. **(C)**: the CT image obtained after horizontal flipping and its corresponding gold standard. **(D)**: the CT image obtained after random cropping and its corresponding gold standard. **(E)**: the CT image obtained after adjusting the contrast and its corresponding gold standard. **(F)**: the CT image obtained after adjusting the brightness and its corresponding gold standard.

CT images have different properties, such as brightness, saturation, and angle. Therefore, a data augmentation method is added in the preprocessing stage of experimental training to prevent overfitting of the training results. In this way, the model performance is increased, and the data augmentation is shown in Figure 10. Figure 10A is the initial image. Figures 10B–F are the corresponding labels of the original image after rotating, horizontally flipping, randomly cropping, adjusting saturation, and adjusting brightness, respectively.

#### 2.5.2 Training parameters

The training set of the CC-CCII dataset is divided into ten groups, each time nine groups of images are used as the training set and one group is used as the validation set. They were used in ten-fold cross-validation experiments. After training and validation separately, we use the test set to test, repeat this process ten times, and finally take the average of the ten results as the evaluation of algorithm accuracy. All CT image pixels are resized to 512 × 512 pixels before being input into the training model. In the model training, the network adopts the mean square error loss function; The initial learning rate of the Adam optimizer is 0.0001; The batch size is set to 64; And the fully connected layer uses a dropout layer with probability 0.5. This deep learning method does not require much analysis of the threshold and gray value of CT images. Data augmentation is achieved by adjusting contrast, affine transformation, and color dithering to achieve better performance of the model. The details of the experimental training parameters are listed in Table 1.

**TABLE 1:** Training parameter settings.

Type	Setting
Batch size	64
Learning rate	0.0001
Optimizer	Adam
Iterations (Epoch)	100
Ubuntu 18.04	PyToch1.6.0

#### 2.5.3 Evaluation indicators

To analyze the segmentation performance of the trained model, we used three common performance metrics: mean intersection over union (mIoU) (Rezatofighi et al., 2019), DSC (Huang et al., 2022), and mean pixel accuracy (mPA) (Paintdakhi et al., 2016). mIoU is the average of the ratios of the intersection and union of the results predicted by the model for each category and the true label, DSC is the similarity measure function, which can calculate the similarity between the true label and the predicted label, and mPA is represents the pixel accuracy of each category. The pixel accuracy is summed and averaged.

$$mIOU=\frac{1}{k+1}\sum_{i=0}^{k}\frac{TP}{TP+FN+FP}$$ (15)

$$DSC=\frac{1}{k+1}\sum_{i=0}^{k}\frac{2TP}{FP+2TP+FN}$$ (16)

$$mPA=\frac{1}{k+1}\sum_{i=0}^{k}\frac{TP}{TP+FN}$$ (17)

where k is the number of classes, TP is the number of pixels that are correctly predicted as positive examples, FN is the number of pixels that are incorrectly predicted as negative examples, and FP is the number of pixels that are incorrectly predicted as positive examples.

## 3 Results and discussion

### 3.1 Ablation experiment

**FIGURE 11:**
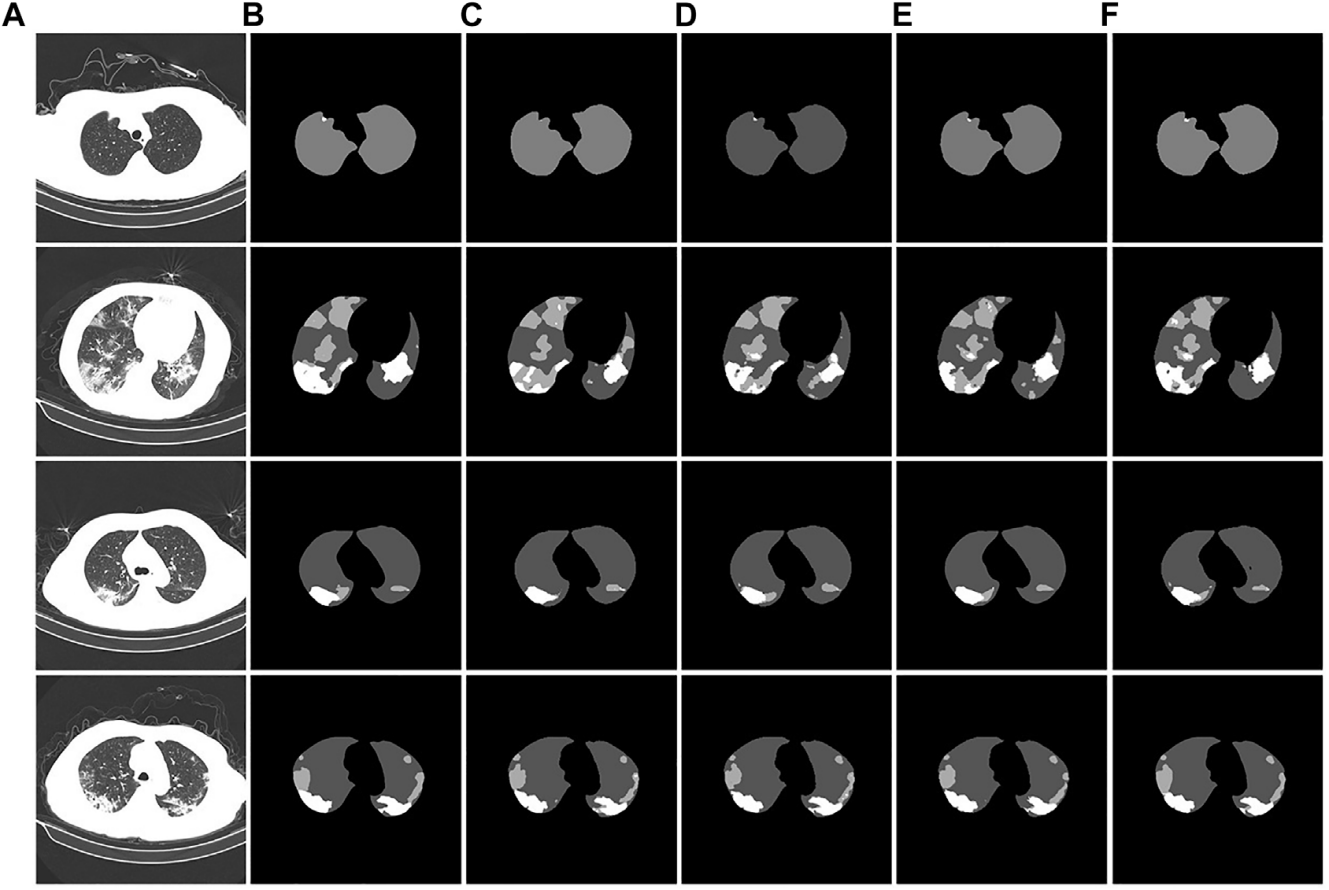
Example of ablation experiment comparison. **(A)**: the CT image of the COVID-19 patient. **(B)**: the gold standard of the CT image. **(C)**: the U-Net segmentation result. **(D)**: the U-Net segmentation result after introducing atrous convolution. **(E)**: the U-Net segmentation result after introducing atrous convolution and CBAM. **(F)**: the U-Net segmentation result after introducing the atrous convolution, CBAM, and Swin Transformer modules.

To verify the segmentation effect of the improved U-Net model, we conducted ablation experiments. The segmentation test results are shown in Figure 11. From the segmentation results of the CT image example, it can be observed that the original U-Net did not segment the tiny lesion details. The other improved models identified the lesions, but the segmentation effects were different. The U-Net segmentation result after adding atrous convolution is shown in Figure 11D. After adding CBAM, the effect is improved, as shown in Figure 11E. The model segmentation results after introducing the atrous convolution, CBAM, and Swin Transformer modules are significantly improved, as shown in Figure 11F. The segmentation performance of our proposed method achieved the best performance; especially in the case of a large number of lesion areas, the segmentation results of lesion and lung areas by this method are closer to the corresponding gold standard.

The experimental segmentation performance indicators are listed in Table 2. On the basis of U-Net, the atrous convolution mPA is added, and the mIoU and DSC indicators are increased by 0.36%, 0.52%, and 0.41%, respectively. After adding atrous convolution and CBAM, the corresponding indicators greatly improved. mPA, mIoU, and DSC metrics improved by 1.55%, 1.82%, and 0.75%, respectively. After adding atrous convolution, the corresponding indicators of CBAM and the Swin Transformer improved the most. The mPA, mIoU, and DSC metrics improved by 1.76%, 2.05%, and 1.53%, respectively. The corresponding metrics demonstrate the effectiveness and feasibility of our method.

**TABLE 2:** Comparison of ablation experiments.

Modle	mPA/%	mIoU/%	DSC/%
U-Net	85.86	78.59	86.74
U-Net + Atrous convolution	86.22	79.11	87.15
U-Net + Atrous convolution + CBAM	87.41	80.41	87.49
U-Net + Atrous convolution + CBAM + Swin Transformer	87.62	80.64	88.27

**FIGURE 12:**
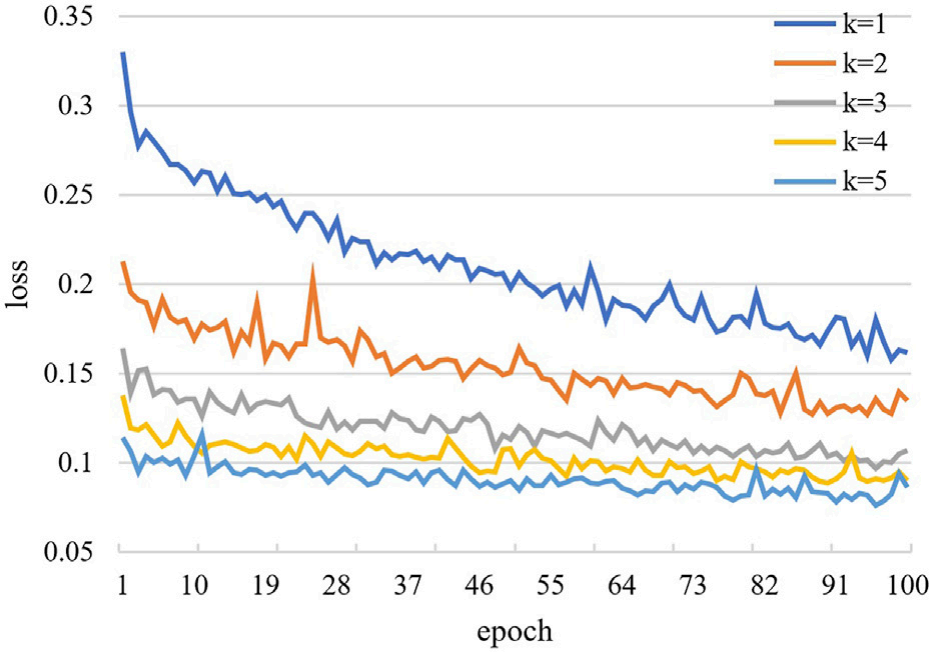
Training convergence loss curve.

The convergence effect of the training loss function of the new model is shown in Figure 12. The curves in the figure represent the training loss curves from the 1st to 5th fold, respectively. After the training method of cross-validation is used, we find that the training loss value of each epoch in fold 1 is the largest and the training loss value of each epoch in fold five is the smallest. The training loss value of each epoch in the next fold is smaller than that of the previous fold. The results show that the convergence effect of the new model is significantly improved.

### 3.2 Models comparison

**FIGURE 13:**
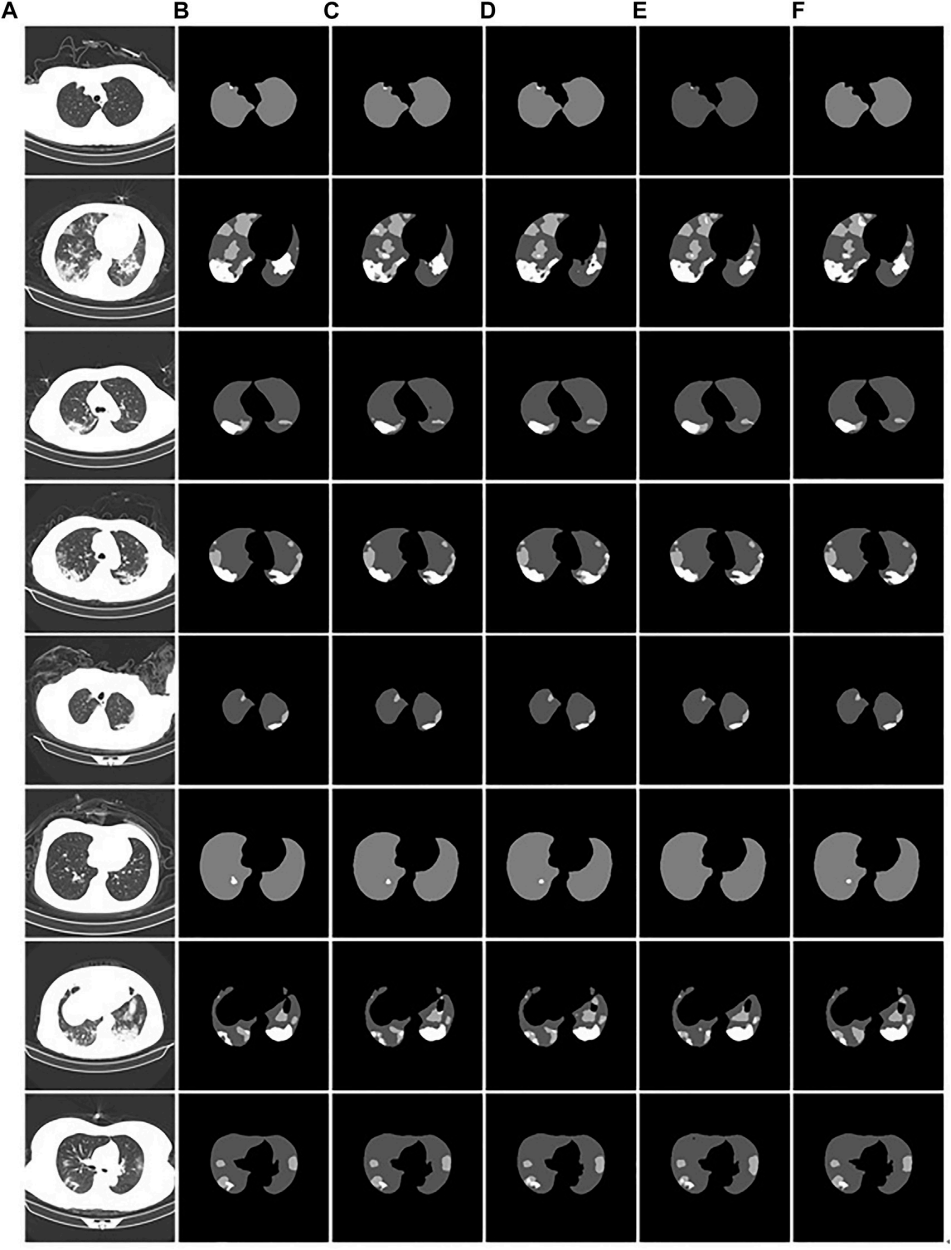
Example of comparison of different models. **(A)**: the CT images of COVID-19 patients. **(B)**: the gold standard of CT images. **(C)**: the segmentation results of our model. **(D)**: the ResU-Net segmentation results. **(E)**: the Attention U-net segmentation results. **(F)**: the TransU-net segmentation result.

We demonstrate the feasibility and effectiveness of the proposed improved method through ablation experiments. To further verify the segmentation ability of the model, we compared it with other models. The results of the segmentation experiment are shown in Figure 13. First, the ResU-Net model (Jha et al., 2019) adds a residual structure to the convolution operations of the encoder and decoder to improve model performance. In CT images of mild patients, our method is compared with the ResU-net method, as shown in Figures 13C,D. In the figure, the performance of the two methods is comparable when segmenting smaller lesions. However, when the proportion of the lesion area is relatively large, the segmentation results show obvious voids, as shown in the second picture in Figure 13D. Second, Attention U-net (Oktay et al., 2018) introduces a soft attention mechanism, which is implemented by supervising the upper-level features through the next-level features. Our method is compared with the attention U-net method, as shown in Figures 13C,E. From the segmentation results, it can be seen that our method performs significantly better than the attention U-net method in terms of lesion segmentation accuracy in smaller regions. Further, leaky segmentation is present in the sixth picture of Figure 13E. Finally, TansU-Net (Chen et al., 2021) applies the Transformer encoder to image segmentation. Our method is compared with the TansU-Net method, as shown in Figures 13C,F. In the segmentation example, the TansU-Net method also appears similar to Attention U-net, failing to successfully identify smaller lesion areas. We used the Swin Transformer encoder structure before the segmentation network decoder. Although CBAM and hole convolution are added, the FLOPs are not much different, and the comprehensive segmentation ability is significantly better than TransU-Net. The effectiveness of our method is further demonstrated, and some complexity is reduced from the Transformer structure.

The comparison performance indicators of the above models are listed in Table 3. The performance metrics of the Attention U-Net method were the worst. The ResU-Net model outperformed TransU-Net in segmentation performance in the used test dataset. Compared with ResU-Net, our proposed segmentation method has improved performance indicators. mPA, mIOU, and DSC were improved by 0.57%, 0.5%, and 0.73%, respectively. Therefore, our proposed method performed the best among the compared models.

**TABLE 3:** Performance comparison of different models.

Model	mPA/%	mIoU/%	DSC/%	FLOPs (G)
ResU-Net	87.05	80.1	87.54	1.46
Attention U-Net	86.26	78.31	86.47	1.95
TransU-Net	86.99	79.33	87.31	1.39
Ours	87.62	80.6	88.27	1.44

### 3.3 Subjective evaluation

For more specialized medical evaluation of segmentation models, clinical validation is required. We invited 10 chief physicians with more than 5 years of clinical experience in radiology to independently perform image analysis (sharpness, resolution, invariance, and acceptability). The scoring criteria for subjective evaluation are shown in Table 4. Ten groups of test samples were randomly constructed, and each group consisted of ten CT images of the lesion area. The subjective quality evaluation results of different algorithms utilized by radiologists are listed in Table 5.

**TABLE 4:** Subjective evaluation scoring method.

Score	Features of the restored image
0	Severely distorted image
1	Image with severe distortion in some areas
2	Slightly distorted image
3	Difficult to spot distorted images
4	Images with better visual effects
5	Very sharp images

**TABLE 5:** Subjective quality evaluation of different algorithms.

Method	Sharpness	Resolution	Invariance	Acceptability
ResU-Net	3.3 ± 0.21	3.5 ± 0.25	0.5 ± 0.39	3.8 ± 0.21
Attention U-Net	3.6 ± 0.24	3.9 ± 0.49	0.6 ± 0.16	3.9 ± 0.41
TransU-Net	3.7 ± 0.16	4.1 ± 0.21	0.6 ± 0.25	4.2 ± 0.24
Our method	3.9 ± 0.24	4.3 ± 0.07	0.7 ± 0.36	4.2 ± 0.81

As shown in Table 5, our proposed Atrous Convolution + CBAM + Swin Transformer model achieves the best subjective quality evaluations in terms of sharpness, resolution, invariance, and acceptability. The main reason is the benefit from introducing W-MSA and the exchange of information. Compared with other segmentation methods, our W-MSA fuses the mutual information and the multimodal features of CT images and has strong representation. The consistency of pathological information between segmented CT image and original CT image was guaranteed. This method achieves the best segmentation effect in terms of ground-glass opacity and visible plaque and lung parenchyma lesions.

## 4 Conclusion

Currently, a key approach to prevent the spread of the epidemic is to combine the chest CT images of patients for diagnosis. Therefore, this paper proposed an improved U-Net network for lesion segmentation in chest CT images of COVID-19. Atrous convolution was used as the convolution operation of each layer of the segmentation network encoder structure, and CBAM was introduced in the downsampling process to solve the problem of loss of lesion detail during the downsampling process. The Swin Transformer module was added to the encoder using the transformer structure to obtain global feature information. The primary improvement of the segmentation model framework is in the encoder part, which improved the model feature extraction performance. The results of the ablation experiments showed that the mPA, mIOU, and DSC reached 87.62, 80.6 and 88.27, respectively. In the subjective evaluation of radiologists, our method can effectively segment ground-glass opacity, visible plaque and lung parenchyma lesions, and maintain consistency with the original CT image pathological information. In future research, we will continue to refine the model. We aim to improve the screening process and the quantitative analysis of the disease and enhancing the efficiency of diagnosis and reducing infection.

## Data Availability

Publicly available datasets were analyzed in this study. This data can be found here: http://ncov-ai.big.ac.cn/download

## References

[B1] Ai T. Yang Z. Hou H. Zhan C. Chen C. Lv W. (2020). Correlation of chest CT and RT-PCR testing for coronavirus disease 2019 (Covid-19) in China: A report of 1014 cases. Radiology 296, E32–E40. 10.1148/radiol.2020200642 32101510 PMC7233399

[B2] Ardila D. Kiraly A. P. Bharadwaj S. Choi B. Reicher J. J. Peng L. (2019). End-to-end lung cancer screening with three-dimensional deep learning on low-dose chest computed tomography. Nat. Med. 25, 954–961. 10.1038/s41591-019-0447-x 31110349

[B3] Bai H. X. Hsieh B. Xiong Z. Halsey K. Choi J. W. Tran T. M. L. (2020). Performance of radiologists in differentiating Covid-19 from non-Covid-19 viral pneumonia at chest CT. Radiology 296 (2), E46–E54. 10.1148/radiol.2020200823 32155105 PMC7233414

[B4] Bernheim A. Mei X. Huang M. Yang Y. Fayad Z. A. Zhang N. (2020). Chest CT findings in coronavirus disease-19 (Covid-19): Relationship to duration of infection. Radiology 295 (3), 200463. 10.1148/radiol.2020200463 32077789 PMC7233369

[B5] Chen J. Lu Y. Yu Q. Luo X. Adeli E. Wang Y. (2021). Transunet: Transformers make strong encoders for medical image segmentation. arXiv: 2102.04306. 10.48550/arXiv.2102.04306

[B6] Chen Y. Li L. (2020). SARS-CoV-2: Virus dynamics and host response. Lancet. Infect. Dis. 20 (5), 515–516. 10.1016/S1473-3099(20)30235-8 32213336 PMC7156233

[B7] Dong E. Du H. Gardner L. M. (2020). An interactive web-based dashboard to track Covid-19 in real time. Lancet. Infect. Dis. 20 (5), 533–534. 10.1016/S1473-3099(20)30120-1 32087114 PMC7159018

[B8] Dosovitskiy A. Beyer L. Kolesnikov A. Weissenborn D. Zhai X. Unterthiner T. (2020). An image is worth 16x16 words: Transformers for image recognition at scale. arXiv. 2010:11929. 10.48550/arXiv.2010.11929

[B9] Esteva A. Kuprel B. Novoa R. A. Ko J. Swetter S. M. Blau H. M. (2017). Dermatologist-level classification of skin cancer with deep neural networks. Nature 542 (7639), 115–118. 10.1038/nature21056 28117445 PMC8382232

[B10] Esteva A. Robicquet A. Ramsundar B. Kuleshov V. DePristo M. Chou K. (2019). A guide to deep learning in healthcare. Nat. Med. 25 (1), 24–29. 10.1038/s41591-018-0316-z 30617335

[B11] Hassani A. Walton S. Shah N. Abuduweili A. Li J. Shi H. (2021). Escaping the big data paradigm with compact transformers. arXiv. 2104:05704. 10.48550/arXiv.2104.05704

[B12] Hatamizadeh A. Nath V. Tang Y. Yang D. Roth H. Xu D. (2022). Swin unetr: Swin transformers for semantic segmentation of brain tumors in mri images. arXiv. 2201:01266.

[B13] Huang K. Xu L. Zhu Y. Meng P. (2022). AU-snake based deep learning network for right ventricle segmentation. Med. Phys. 49 (6), 3900–3913. 10.1002/mp.15613 35302251

[B14] Jaiswal A. Gianchandani N. Singh D. Kumar V. Kaur M. (2020). Classification of the Covid-19 infected patients using DenseNet201 based deep transfer learning. J. Biomol. Struct. Dyn. 39 (15), 5682–5689. 10.1080/07391102.2020.1788642 32619398

[B15] Jha D. Smedsrud P. H. Riegler M. A. Johansen D. Lange T. D. Halvorsen P. (2019). “Resunet++: An advanced architecture for medical image segmentation.” in Proceedings IEEE International Symposium on Multimedia (ISM). (San Diego, CA, USA: IEEE), 225–2255. 10.1109/ISM46123.2019.00049

[B16] Lee Y. Hara T. Fujita H. Itoh S. Ishigaki T. (2001). Automated detection of pulmonary nodules in helical CT images based on an improved template-matching technique. IEEE Trans. Med. Imaging 20 (7), 595–604. 10.1109/42.932744 11465466

[B17] Li L. Qin L. Xu Z. Yin Y. Wang X. Kong B. (2020). Using artificial intelligence to detect COVID-19 and community-acquired pneumonia based on pulmonary CT: Evaluation of the diagnostic accuracy. Radiology 296, E65–E71. 200905. 10.1148/radiol.2020200905 32191588 PMC7233473

[B18] Litjens G. Kooi T. Bejnordi B. E. Setio A. A. A. Ciompi F. Ghafoorian M. (2017). A survey on deep learning in medical image analysis. Med. Image Anal. 42, 60–88. 10.1016/j.media.2017.07.005 28778026

[B19] Liu Z. Lin Y. Cao Y. Hu H. Wei Y. Zhang Z. (2021). Swin transformer: Hierarchical vision transformer using shifted windows. In.Proceedings of the IEEE/CVF International Conference on Computer Vision, 10012–10022. 10.48550/arXiv.2103.14030

[B20] Mei X. Lee H. C. Diao K. Huang M. Lin B. Liu C. (2020). Artificial intelligence–enabled rapid diagnosis of patients with Covid-19. Nat. Med. 26 (8), 1224–1228. 10.1038/s41591-020-0931-3 32427924 PMC7446729

[B21] Oktay O. Schlemper J. Folgoc L. L. Lee M. Heinrich M. Misawa K. (2018). Attention u-net: Learning where to look for the pancreas. arXiv:1804.03999. 10.48550/arXiv.1804.03999

[B22] Paintdakhi A. Parry B. Campos M. Irnov I. Elf J. Surovtsev I. (2016). Oufti: An integrated software package for high-accuracy high-throughput quantitative microscopy analysis. Mol. Microbiol. 99 (4), 767–777. 10.1111/mmi.13264 26538279 PMC4752901

[B23] Qin Z. Z. Sander M. S. Rai B. Titahong C. N. Sudrungrot S. Laah S. N. (2019). Using artificial intelligence to read chest radiographs for tuberculosis detection: A multi-site evaluation of the diagnostic accuracy of three deep learning systems. Sci. Rep. 9 (1), 15000. 10.1038/s41598-019-51503-3 31628424 PMC6802077

[B24] Rezatofighi H. Tsoi N. Gwak J. Sadeghian A. Reid I. Savarese S. (2019). “Generalized intersection over union: A metric and a loss for bounding box regression.” in Proceedings of the IEEE/CVF conference on computer vision and pattern recognition, 15-20 June 2019. (Long Beach, CA, USA: IEEE), 658–666. 10.1109/CVPR.2019.00075

[B25] Rubin G. D. Ryerson C. J. Haramati L. B. Sverzellati N. Kanne J. P. Raoof S. (2020). The role of chest imaging in patient management during the covid-19 pandemic: A multinational consensus statement from the fleischner society. Chest 296 (1), 106–116. 10.1016/j.chest.2020.04.003 PMC7138384 32275978

[B26] Shi H. Han X. Jiang N. Cao Y. Alwalid O. Gu J. (2020). Radiological findings from 81 patients with covid-19 pneumonia in wuhan, China: A descriptive study. Lancet. Infect. Dis. 20 (4), 425–434. 10.1016/S1473-3099(20)30086-4 32105637 PMC7159053

[B27] Song Y. Zheng S. Li L. Zhang X. Zhang X. Huang Z. (2021). Deep learning enables accurate diagnosis of novel coronavirus (COVID-19) with CT images. IEEE/ACM Trans. Comput. Biol. Bioinform. 18 (6), 2775–2780. 10.1109/TCBB.2021.3065361 33705321 PMC8851430

[B28] Topol E. J. (2019). High-performance medicine: The convergence of human and artificial intelligence. Nat. Med. 25 (1), 44–56. 10.1038/s41591-018-0300-7 30617339

[B29] Valanarasu J. M. J. Oza P. Hacihaliloglu I. Patel V. M. (2021). Medical transformer: Gated axial-attention for medical image segmentation. arXiv. 2102:10662. 10.1007/978-3-030-87193-2_4

[B30] Wang H. Cao P. Wang J. Zaiane O. R. (2021). Uctransnet: Rethinking the skip connections in u-net from a channel-wise perspective with transformer. arXiv:2109.04335. 10.48550/arXiv.2109.04335

[B31] Wang H. Zhu Y. Green B. Adam H. Yuille A. Chen L. C. (2020). “Axial-deeplab: Stand-alone axial-attention for panoptic segmentation.” in Proceedings of the European conference on computer vision (ECCV), 29 October 2020. Berlin, Germany: Springer, 108–126. 10.1007/978-3-030-58548-8_7

[B32] Wong H. Y. F. Lam H. Y. S. Fong A. H. T. Leung S. T. Chin T. W. Y. Lo C. S. Y. (2020). Frequency and distribution of chest radiographic findings in patients positive for Covid-19. Radiology 296 (2), E72–E78. 10.1148/radiol.2020201160 32216717 PMC7233401

[B33] Woo S. Park J. Lee J. Y. Kweon I. S. (2018). “Cbam: Convolutional block attention module.” in Proceedings of the European conference on computer vision (ECCV), 06 October 2018. Cham: Springer, 3–19. 10.1007/978-3-030-01234-2_1

[B34] Xu X. Jiang X. Ma C. Du P. Li X. Lv S. (2020). A deep learning system to screen novel coronavirus disease 2019 pneumonia. Engineering 6 (10), 1122–1129. 10.1016/j.eng.2020.04.010 32837749 PMC7320702

[B35] Zhu N. Zhang D. Wang W. Li X. Yang B. Song J. (2019). A novel coronavirus from patients with pneumonia in China, 2019. N. Engl. J. Med. 382, 727–733. 10.1056/NEJMoa2001017 PMC7092803 31978945

